# When Histology Is Unknown: Surgical Decision-Making in Small Bowel Tumors—From Preoperative Imaging to Intraoperative Findings: A Narrative Review

**DOI:** 10.3390/jcm15187106

**Published:** 2026-09-13

**Authors:** Cosmin Vasile Obleaga, Sergiu Marian Cazacu, Cecil Sorin Mirea, Radu Mesina, Alexandru Marin Pascu, Georgica Costinel Târtea, Mirela-Marinela Florescu, Dragos-Florin Vasile, Lucian Mihai Florescu, Ion Vasile, Cristin Constantin Vere

**Affiliations:** 1 Department of Surgical Oncology, University of Medicine and Pharmacy of Craiova, 200349 Craiova, Romaniacecil.mirea@umfcv.ro (C.S.M.); mesinaradu@yahoo.com (R.M.);; 2 Research Center of Gastroenterology and Hepatology, University of Medicine and Pharmacy of Craiova, 200349 Craiova, Romania; 3 Department of Cardiology, University of Medicine and Pharmacy of Craiova, 200349 Craiova, Romania; georgica.tartea@umfcv.ro; 4 Department of Pathology, University of Medicine and Pharmacy of Craiova, 200349 Craiova, Romania; 5 Department of Urology, University of Medicine and Pharmacy of Craiova, 200349 Craiova, Romania; dragos.vasile@umfcv.ro; 6 Department of Radiology and Medical Imaging, University of Medicine and Pharmacy of Craiova, 200349 Craiova, Romania; 7 Dolj Section, Romanian Academy of Medical Sciences, Craiova Branch, 200349 Craiova, Romania

**Keywords:** small bowel tumor, small bowel neoplasm, surgical decision-making, computed tomography, intraoperative findings, SBNET, device-assisted enteroscopy

## Abstract

Small-bowel tumors are a heterogeneous and relatively uncommon group of neoplasms whose definitive diagnosis frequently cannot be established before surgery. Their common jejunal or ileal location limits conventional endoscopic access, and stenosing, predominantly extraluminal, or complicated presentations may render preoperative biopsy impossible. In such situations, the surgeon may have to decide the extent of resection and the need for lymphadenectomy without a histological diagnosis, even though the optimal strategy differs substantially among adenocarcinoma, gastrointestinal stromal tumor (GIST), small-bowel neuroendocrine tumor (SBNET), lymphoma, and desmoid-type fibromatosis. This narrative review integrates clinical presentation, preoperative computed tomography (CT) features, and intraoperative macroscopic findings to support a presumptive diagnosis and guide surgical decision-making when histology is unavailable. The evidence was synthesized along the decision pathway preoperative imaging → diagnostic suspicion → intraoperative appearance → surgical decision → definitive histopathology. Histology-specific CT associations are relatively well documented, whereas the independent ability of intraoperative macroscopic impression to predict histology remains limited. We propose a pragmatic, histology-oriented decision framework that prioritizes clinical urgency, disease extent and resectability, the feasibility of preoperative tissue sampling, and the potential impact of histology on the surgical procedure. The framework is conceptual and requires prospective validation; its aim is not to replace histopathological examination but to reduce the risk of both oncologically inadequate and unnecessarily extensive resections.

## 1. Introduction

Small-bowel tumors represent a heterogeneous group of neoplastic lesions characterized by considerable histopathological diversity and by an incidence markedly lower than that of neoplasms arising in other gastrointestinal segments; although very rare, the incidence has increased in recent decades, with 1.4/100,000 inhabitants in North America and Europe, and 2.3/100,000 people (0.6% of all cancer diagnoses) in the USA in 2018 [1,2]. The principal malignant entities are adenocarcinoma, small-bowel neuroendocrine tumors (SBNETs), gastrointestinal stromal tumors (GISTs), and lymphomas, alongside benign tumors, mesenchymal lesions, and tumors of intermediate biological behavior—such as desmoid-type fibromatosis—that may also be encountered [1,3].

The diagnosis of small-bowel tumors remains difficult. Symptoms are frequently nonspecific and include abdominal pain, anemia, gastrointestinal bleeding, weight loss, or altered bowel transit; some tumors become clinically apparent only when a complication such as intestinal obstruction, perforation, hemorrhage, or intussusception develops [1,2]. Moreover, the frequently jejunal or ileal location limits accessibility to conventional digestive endoscopy, so obtaining a preoperative histopathological diagnosis may be difficult and may require advanced techniques such as device-assisted enteroscopy (DAE) [4].

The development of CT and MR enterography, video capsule endoscopy, and DAE has considerably improved diagnostic capability [5,6]. Nevertheless, even advanced endoscopic techniques do not guarantee a histopathological diagnosis in all cases. A stenosing lesion, a difficult-to-access location, predominantly extraluminal growth, the risk of hemorrhage or perforation, and presentation in an emergency setting may make it impossible to obtain a biopsy before surgical treatment [7]. Even with double-balloon enteroscopy, endoscopic biopsy yielded a histopathological diagnosis in 59.5% of mass-like lesions, whereas in 40.5% of cases the definitive diagnosis was established only after surgery [4]. In these situations, preoperative imaging becomes essential not only for identifying and staging the tumor but also for guiding the differential diagnosis. Lesion morphology, a circumferential or exophytic pattern, the degree of stenosis, the contrast-enhancement pattern, and the presence of necrosis, lymphadenopathy, mesenteric changes, or secondary deposits may suggest a particular histological origin [8,9].

Knowledge of the histopathological type may fundamentally alter the surgical strategy [10,11], while the molecular heterogeneity reported in gastrointestinal adenocarcinomas further illustrates the biological complexity underlying tumor behavior [12,13]. In small-bowel adenocarcinoma, oncological resection entails removal of the affected intestinal segment together with its mesentery and regional lymph nodes [3]. In GIST, the principal objective is complete resection while avoiding tumor rupture, whereas systematic lymphadenectomy is not routinely required [14]. In SBNET, multifocality, nodal metastases, and mesenteric fibrosis mandate careful exploration of the bowel and a specific strategy toward the mesentery and regional lymphatic territory [15,16]. For lymphoma, the role of surgery differs and is significantly influenced by complications such as obstruction, perforation, or hemorrhage [17]. At the same time, an apparently malignant mesenteric or intestinal mass may represent desmoid-type fibromatosis, in which extensive resection performed with oncological intent may not always be the optimal strategy [18].

Thus, the same clinical presentation—a small-bowel mass without a preoperative histopathological diagnosis—may conceal diseases for which the surgical principles are profoundly different [10]. The surgeon may be forced to decide intraoperatively whether to perform a simple biopsy, a segmental resection, an oncological resection with lymphadenectomy, or, conversely, to avoid an extensive procedure until a definitive diagnosis is available. In this context, preoperative clinico-imaging data and intraoperative macroscopic appearance represent additional sources of information. The tumor growth pattern, an infiltrative or expansile character, the relationship to the mesentery, the distribution and appearance of lymphadenopathy, multifocality, the presence of mesenteric fibrosis or retraction, invasion of adjacent organs, and possible metastatic deposits may all orient and modify the diagnostic suspicion formulated preoperatively [1,8,9,19]. These elements cannot substitute for histopathological examination, but they may decisively influence operative management when histology is not available [1,2,4,19].

Most existing guidelines and reviews address adenocarcinoma, GIST, SBNET, lymphomas, or desmoid tumors separately, generally starting from an already established histological diagnosis. Far less frequently discussed is the reverse situation, commonly encountered in surgical practice: the tumor is known and the operative indication exists, but its histological nature remains uncertain at the time of surgery. The objective of this study is therefore to analyze surgical decision-making in small-bowel tumors without a preoperative histopathological diagnosis, integrating information provided by the clinical picture, preoperative imaging, and intraoperative macroscopic findings. The principal differential diagnoses—adenocarcinoma, GIST, SBNET, lymphomas, and desmoid-type fibromatosis—are analyzed, with emphasis on the features that may orient the diagnosis and, above all, on how diagnostic suspicion should influence the extent of resection, lymphadenectomy, and the indication for biopsy. Finally, we integrate these elements into a practical decision algorithm intended for situations in which the surgeon must choose an operative strategy before the definitive histopathological diagnosis is obtained.

## 2. Materials and Methods

A structured narrative review of the literature was performed to identify relevant studies on the diagnosis and surgical management of small-bowel tumors in situations in which a preoperative histopathological diagnosis is absent or uncertain. The analysis focused specifically on the role of clinical, imaging (CT), and intraoperative macroscopic features in orienting the differential diagnosis and selecting the surgical strategy.

**Figure 1:**
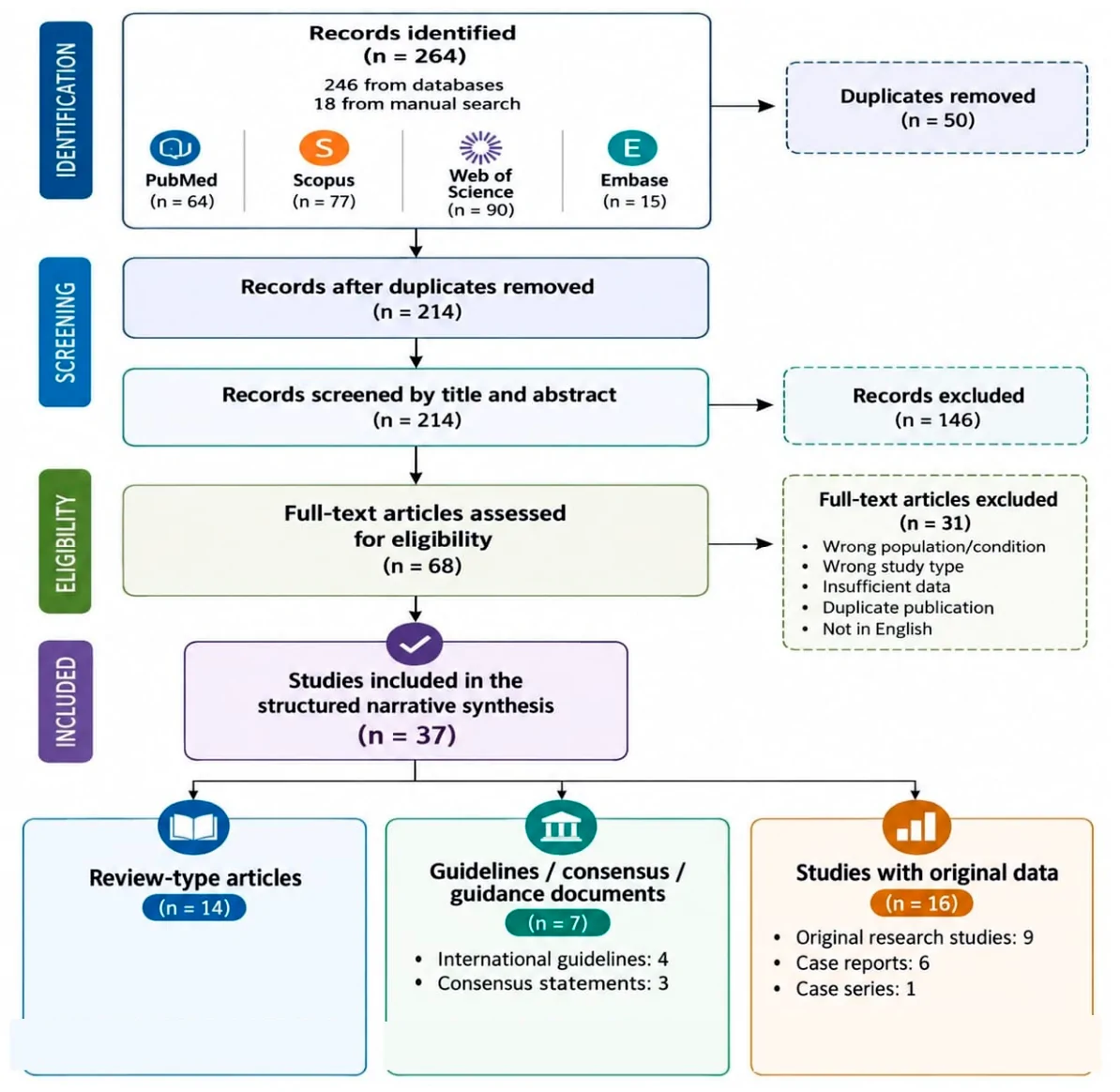
Literature identification and selection process. Note: Google Scholar was used only as a supplementary/manual search source and was not counted as a primary database in the numerical flow. References for the review-type articles group: [6,7,9,20,21,23,24,25,27,29,30,38,43,44]; for the guidelines/consensus documents group: [14,16,18,28,31,32,36]; for the studies-with-original-data group: [1,2,4,10,17,22,26,33,34,35,37,39,40,41,42,45].

A structured search of PubMed, Scopus, Web of Science, and Embase was conducted for studies published between January 2020 and August 2026. Google Scholar was used only as a supplementary source to identify additional relevant publications and was not treated as a primary database for the numerical study-selection flow. The final database search across all sources was completed on 03 August 2026. The study identification and selection process is summarized in Figure 1.

The search strategy combined keywords and controlled vocabulary, including MeSH terms, such as: “small bowel tumor,” “small bowel neoplasm,” “small intestinal tumor,” “small bowel cancer,” “small bowel adenocarcinoma,” “gastrointestinal stromal tumor,” “GIST,” “small bowel neuroendocrine tumor,” “intestinal lymphoma,” “desmoid tumor,” “mesenteric desmoid,” “preoperative diagnosis,” “histological diagnosis,” “computed tomography,” “CT enterography,” “MR enterography,” “capsule endoscopy,” “device-assisted enteroscopy,” “intraoperative findings,” “macroscopic appearance,” “surgical decision-making,” “surgical management,” “bowel resection,” and “lymphadenectomy.”

The literature search was carried out in two stages. In the first stage, general studies on the diagnosis, imaging features, and surgical management of small-bowel tumors were identified. In the second stage, targeted searches were performed for the principal histopathological entities relevant to the differential diagnosis and surgical decision-making.

Eligibility criteria were: (1) guidelines, consensus documents, cohort studies, case series, case reports, diagnostic-performance studies, and review articles relevant to small-bowel tumor diagnosis, imaging, intraoperative findings, or surgical strategy; (2) studies addressing small-bowel tumors or tumor-like lesions relevant to the decision pathway; and (3) publications providing sufficient information to assess the diagnostic or therapeutic implications. Exclusion criteria were: (1) publications in languages other than English; (2) studies without clinical or surgical relevance to the review aim; (3) exclusively experimental studies; (4) publications for which the available information did not allow assessment of diagnosis or therapeutic strategy; and (5) studies reporting overlapping populations or duplicate datasets.

Title/abstract screening and full-text assessment were performed independently by two authors (C.V.O. and C.C.V.). Disagreements were resolved by discussion and, when necessary, by adjudication of a third author (S.M.C.).

The analysis was structured around the decision pathway relevant to surgical practice: preoperative imaging → diagnostic suspicion → intraoperative macroscopic appearance → surgical decision → definitive histopathological diagnosis. Studies published before 2020 were used only as background/context references (e.g., definitions, foundational imaging descriptions, or historical guidelines) and were not included in the formal 2020–August 2026 structured synthesis.

Given the narrative character of the review and the considerable heterogeneity of the included studies with respect to design, population, histopathological type, and analyzed objectives, a formal risk-of-bias assessment was not performed, and no quantitative synthesis or meta-analysis was undertaken.

Of the 37 studies included in the structured evidence synthesis below (Table 1a–c), 3 were published in 2020 [6,20,21], 1 in 2021 [22], 9 in 2022 [9,10,14,23,24,25,26,27,28], 6 in 2023 [4,29,30,31,32,33], 7 in 2024 [2,7,16,18,34,35,36], 5 in 2025 [1,17,37,38,39], and 6 in 2026 [40,41,42,43,44,45], reflecting the recency of the evidence base for this topic. Within each of the three tables below, studies are grouped by category and ordered chronologically for readability rather than by reference number.

## 3. Epidemiology and Impact

Small-bowel tumors account for approximately 3–6% of gastrointestinal neoplasms [4] and are characterized by important histopathological heterogeneity and frequently delayed diagnosis. Recent data from a tertiary center in Romania confirm this feature: in a series of 80 patients with small-bowel tumors, 72.5% of the lesions were malignant, adenocarcinoma being the most frequent primary malignant tumor [1]. Despite advances in endoscopic methods, CT remains one of the principal means of identifying these tumors, particularly for bulky lesions and in patients presenting as emergencies [1,2]. In a Romanian surgical series of 46 resectable primary small-bowel cancers, CT performed in the emergency department identified the tumor in 15 of the 16 investigated patients (93.8%), and the increasing use of CT after 2020 led to a higher proportion of tumors diagnosed preoperatively than in the earlier period, when complicated forms were more often diagnosed intraoperatively [2].

The impact of these tumors on surgical decision-making, however, is determined not only by their frequently late or complicated presentation but above all by the possible absence of a histopathological diagnosis at the time of surgery. Patients operated on for obstruction, perforation, or hemorrhage may require intervention before a biopsy can be obtained, and in elective settings a difficult-to-access location, a stenosing or predominantly extraluminal tumor, and the incomplete yield of endoscopic biopsy may lead to the same problem [4,7,30]. The surgeon may therefore be obliged to decide the extent of resection and the need for lymphadenectomy without knowing the definitive histological nature of the lesion.

In this context, CT acquires an additional role, beyond simple identification and staging, because certain morphological features may orient the histological diagnosis. Recent radiological-pathological studies have demonstrated associations between CT appearance and definitive histology, such as irregular/asymmetric wall thickening for adenocarcinoma, exophytic growth for GIST, lymphadenopathy with relative luminal preservation for lymphoma, and mesenteric changes for SBNET [22,29,38]. Nevertheless, this predictive capacity remains imperfect, and intraoperative macroscopic findings must be interpreted as complementary information rather than as a substitute for histopathological examination. Consequently, the relevant question for the surgeon is not merely the identification of a small-bowel tumor, but the estimation of the probability of a given histological diagnosis by integrating CT data with intraoperative appearance, so that the surgical strategy chosen in the absence of definitive histology is oncologically adequate while avoiding an unjustifiably extensive procedure [17,35,37].

From the surgeon’s perspective, this diagnostic uncertainty becomes particularly relevant when the histological nature of the tumor cannot be established preoperatively, because a strategy not adapted to the definitive histology may lead either to an oncologically insufficient procedure or to an unjustifiably extensive resection [29,30].

## 4. Tumor Types (Clinical, Imaging, and Intraoperative Appearance)

### 4.1. Adenocarcinoma

Small-bowel adenocarcinoma frequently exhibits an infiltrative, stenosing growth pattern, a feature that directly influences its clinical presentation. Unlike predominantly exophytic tumors, the lesion need not reach a large size to become symptomatic, because circumferential infiltration of the wall and progressive luminal narrowing may cause early transit disturbances and, subsequently, intestinal obstruction. In a recent review, approximately 60% of patients with small-bowel adenocarcinoma were symptomatic at presentation, stenosis-related manifestations being the most frequent; symptomatic presentation was reported more often for jejunoileal than for duodenal locations (84% versus 54%) [23].

Clinically, intermittent colicky abdominal pain, nausea, and vomiting may reflect the progressive onset of obstruction, whereas anemia or gastrointestinal bleeding may result from tumor ulceration. Jejunoileal forms are particularly relevant to the surgeon, because the diagnosis may be established only after a complication develops, obstruction being one of the characteristic modes of presentation [23,31].

The CT appearance reflects this biological growth pattern. Jejunal or ileal adenocarcinoma typically appears as focal or segmental, irregular and asymmetric—sometimes circumferential—wall thickening associated with abrupt luminal narrowing. The lesion may have an annular, stenosing, apple-core appearance, and dilatation of the proximally located loops is an important indicator of its obstructive character [23,29]. Contrast enhancement is generally moderate and may be heterogeneous. In advanced forms, infiltration of the adjacent mesentery, regional lymphadenopathy, vascular invasion, peritoneal carcinomatosis, or hepatic metastases may be identified [23,42]. These features are also useful in the differential diagnosis. In the radiological–pathological study by Bayoumi et al., irregular/asymmetric wall thickening was identified in 14 of the 15 adenocarcinomas constituting one of the features significantly associated with histology [22]. Compared with GIST, adenocarcinoma more frequently shows an infiltrative, stenosing pattern, whereas exophytic growth is more characteristic of GIST; compared with lymphoma, adenocarcinoma more frequently causes irregular luminal narrowing and intestinal obstruction [29,46].

**Figure 2:**
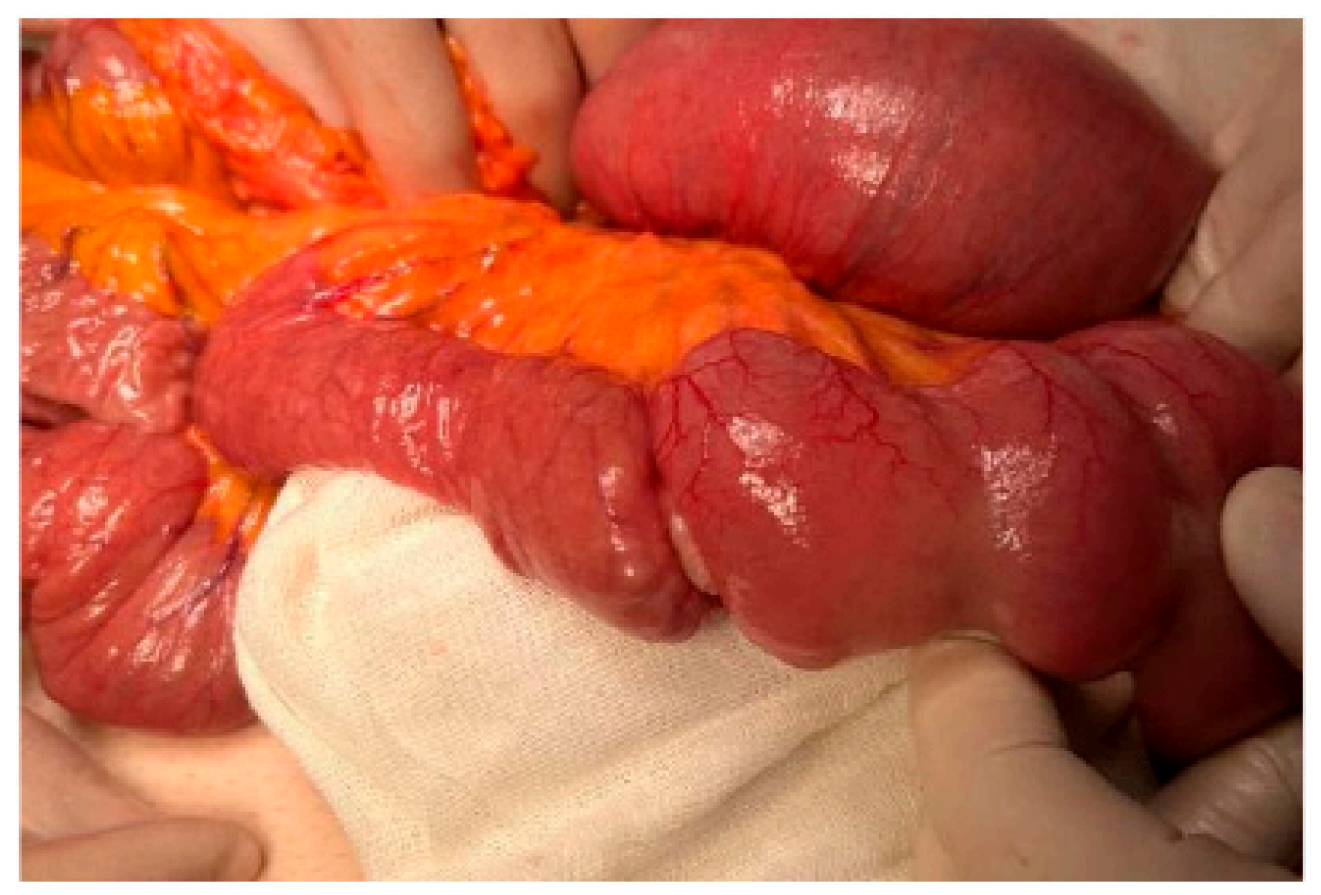
Intraoperative appearance of adenocarcinoma (suspected on CT and intraoperatively; confirmed histopathologically). Original intraoperative image from the authors’ own material, not previously published.

With respect to intraoperative assessment, this growth pattern may translate into an infiltrative, circumferential or semicircumferential parietal lesion with a stenosing character and variable dilatation of the proximal loop (Figure 2). Transmural extension may involve the serosa and the adjacent mesentery, and the presence of regional lymphadenopathy may provide an additional diagnostic clue [23,47]. However, these findings are not pathognomonic, and in complicated forms associated inflammatory changes, perforation, or invasion of adjacent structures may modify the macroscopic appearance and hinder intraoperative assessment. In the analyzed literature, correlations between CT features and histopathological diagnosis are well documented [22,29], whereas data on the ability of the intraoperative macroscopic appearance to independently predict the histological type are more limited.

Immediate macroscopic examination of the resection specimen may provide additional information on the tumor growth pattern. Jejunoileal adenocarcinoma may show an infiltrative, ulcerated, or annular-stenosing appearance, with circumferential involvement of the wall and evident luminal reduction [23,42]. This correspondence is illustrated by Lee et al., who present a jejunal adenocarcinoma with abrupt luminal narrowing and proximal intestinal dilatation on CT, corresponding to a stenosing lesion on the operative specimen and subsequently confirmed histopathologically as adenocarcinoma [29]. Thus, in a patient operated on without a preoperative histopathological diagnosis, the association of an obstructive presentation, a short, irregular, stenosing CT lesion, an infiltrative circumferential intraoperative appearance, and possible regional lymphadenopathy may support the suspicion of adenocarcinoma and contribute to selecting a resection consistent with oncological principles.

### 4.2. Gastrointestinal Stromal Tumor (GIST)

Unlike adenocarcinoma, small-bowel gastrointestinal stromal tumors frequently exhibit an exophytic growth pattern, developing predominantly toward the peritoneal cavity and causing significant luminal narrowing relatively late. Consequently, GISTs may reach considerable dimensions before obstructive symptoms appear, and the clinical picture is influenced mainly by tumor size, location, and the occurrence of complications. Exophytic and mixed patterns are the most frequently described growth forms for small-bowel GISTs [48].

Clinically, manifestations may be nonspecific, including abdominal pain or discomfort, distension, and anemia, whereas gastrointestinal bleeding is one of the characteristic presentations and is frequently associated with ulceration of the overlying mucosa. Intestinal obstruction may occur, but the mechanism differs from the infiltrative stenosing character typical of adenocarcinoma: it may be caused by the endoluminal component, by compression from an exophytic mass, or, more rarely, by intussusception or intestinal torsion associated with a bulky tumor. Tumor rupture, intraperitoneal hemorrhage, and perforation are other possible modes of acute presentation [24,40].

The CT appearance of small-bowel GIST reflects its predominantly extraluminal development. The lesion typically appears as a well-delineated, exophytic mass arising from the intestinal wall, with variable contrast enhancement. Small tumors tend to be relatively homogeneous and well circumscribed, whereas bulky lesions frequently become heterogeneous owing to central necrosis, intratumoral hemorrhage, or cavitation. Calcifications may be present but are less frequent. In a radiological review, necrosis was reported in approximately 39% of small-bowel GISTs, the proportion rising to 66.7–100% for tumors ≥ 5 cm; intratumoral hemorrhage was described in 66.7–88.9% of tumors of these dimensions [48].

This morphology provides important elements for the differential diagnosis. In the radiological–pathological study by Bayoumi et al., exophytic growth was identified in all 11 analyzed GISTs, representing the imaging feature most strongly associated with this histology [22]. Unlike adenocarcinoma, GIST less frequently causes irregular circumferential luminal narrowing and proximal obstruction, and regional lymphadenopathy is unusual, which constitutes an important differentiating element both from adenocarcinoma and, especially, from lymphoma [29].

**Figure 3:**
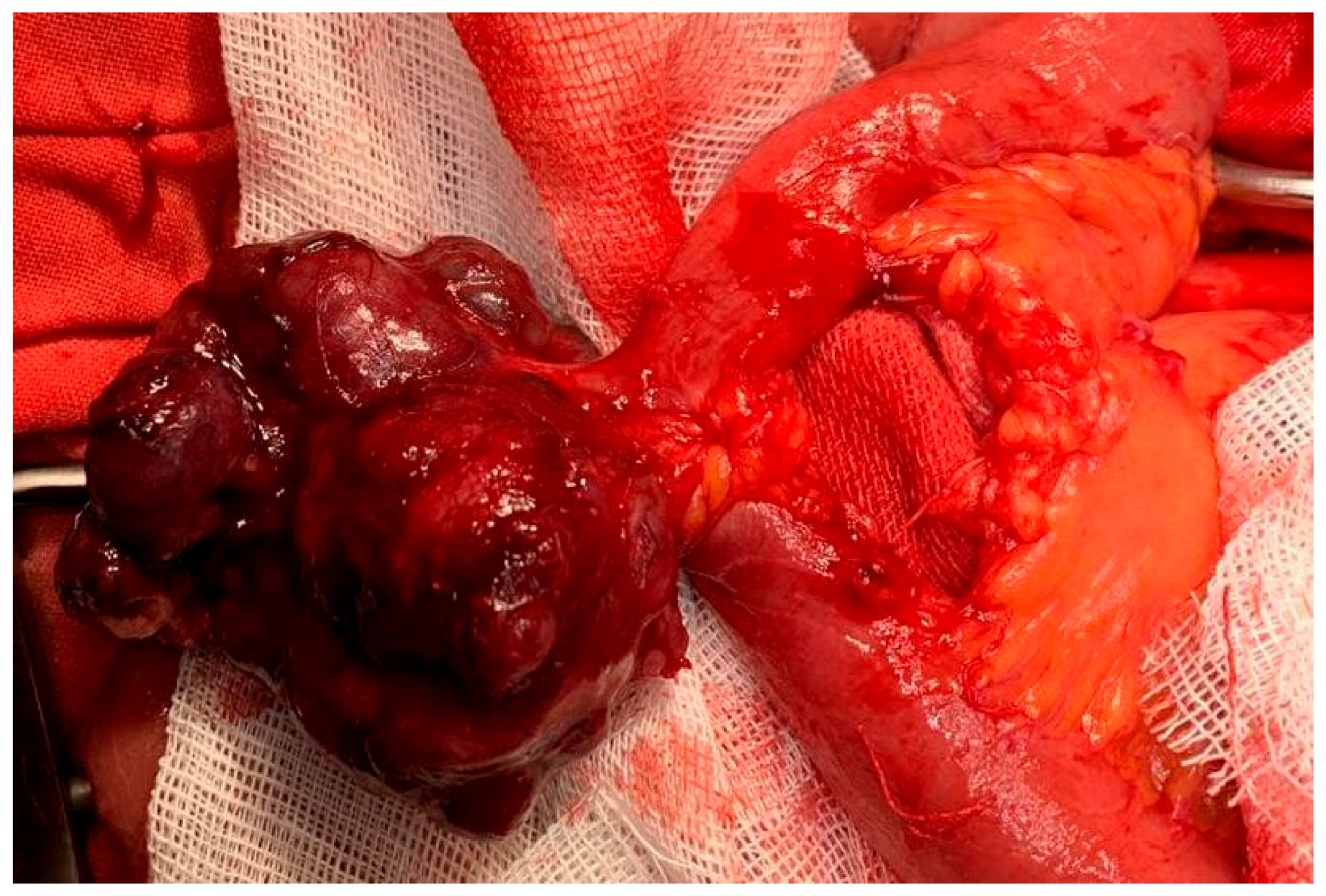
Intraoperative appearance of GIST (suspected on CT and intraoperatively; confirmed by histopathology and immunohistochemistry). Original intraoperative image from the authors’ own material, not previously published.

Intraoperatively, small-bowel GIST may present as a well-delineated, predominantly exophytic mass arising from the intestinal wall and covered by a pseudocapsule, sometimes with an associated intraluminal component (Figure 3). Bulky tumors may show areas of necrosis, hemorrhage, or cystic degeneration and may adhere to intestinal loops, the mesentery, or adjacent organs, without these relationships necessarily signifying tumor invasion. In the recent series by Bist et al., intraoperative findings ranged from bulky exophytic jejunal or ileal masses to intraluminal lesions and perforated necrotic exophytic forms [40]. The absence of significant regional lymphadenopathy in association with a well-delineated exophytic mass may constitute an additional element orienting intraoperatively toward GIST, although the macroscopic appearance is not pathognomonic. A particularly important feature for the surgeon is the integrity of the pseudocapsule, because excessive manipulation and tumor rupture must be avoided. Contemporary guidelines recommend, for localized GIST, complete R0 resection with preservation of an intact pseudocapsule and do not recommend systematic lymphadenectomy in the absence of macroscopically suspicious lymph nodes, except for particular subtypes [14,32].

On immediate macroscopic examination of the resection specimen, GIST may appear as a nodular or lobulated, well-circumscribed formation with predominantly extraluminal development and a solid, grayish-white cut surface. Large tumors may show central areas of hemorrhage, necrosis, or cystic degeneration, sometimes communicating with the intestinal lumen through mucosal ulceration [32,49]. Thus, in a patient operated on without a preoperative histopathological diagnosis, the association of a predominantly exophytic mass on CT, extraluminal development confirmed intraoperatively, absence of significant regional lymphadenopathy, and the presence of a pseudocapsule may support the suspicion of GIST. This suspicion has a direct surgical consequence: it favors complete resection with preservation of tumor integrity and avoidance of rupture, without systematic lymphadenectomy in the absence of suspicious nodes [22,29,32,50].

### 4.3. Lymphoma

Primary small-bowel lymphoma exhibits a growth pattern different from that of adenocarcinoma, frequently characterized by circumferential infiltration of the intestinal wall over a relatively long segment, without a marked desmoplastic reaction. Consequently, even lesions with pronounced wall thickening may cause less severe stenosis than adenocarcinoma. Infiltration of the muscularis and of the nerve plexuses may lead to loss of parietal tone and aneurysmal dilatation of the affected intestinal segment, an appearance suggestive of lymphoma [29]. Unlike adenocarcinoma, dilatation of the intestinal loops proximal to the tumor may be absent even in the presence of a large lesion. In the radiological–pathological study by Bayoumi et al., absence of proximal intestinal dilatation was observed in 10 of the 13 lymphomas [22].

Clinically, manifestations are variable and often nonspecific, including abdominal pain, weight loss, anemia, gastrointestinal bleeding, nausea, or vomiting. Small-bowel lymphoma may, however, become a surgical problem through the occurrence of complications, particularly perforation, hemorrhage, or intestinal obstruction. In these situations, inflammatory changes and those secondary to perforation or obstruction may dominate the clinical and imaging picture, partly masking the morphological features of the tumor. Cases in the literature confirm that an acute complication may even be the initial mode of diagnosis of a gastrointestinal lymphoma—either through perforation in jejunoileal locations or through severe gastrointestinal bleeding in gastric locations [41,51].

**Figure 4:**
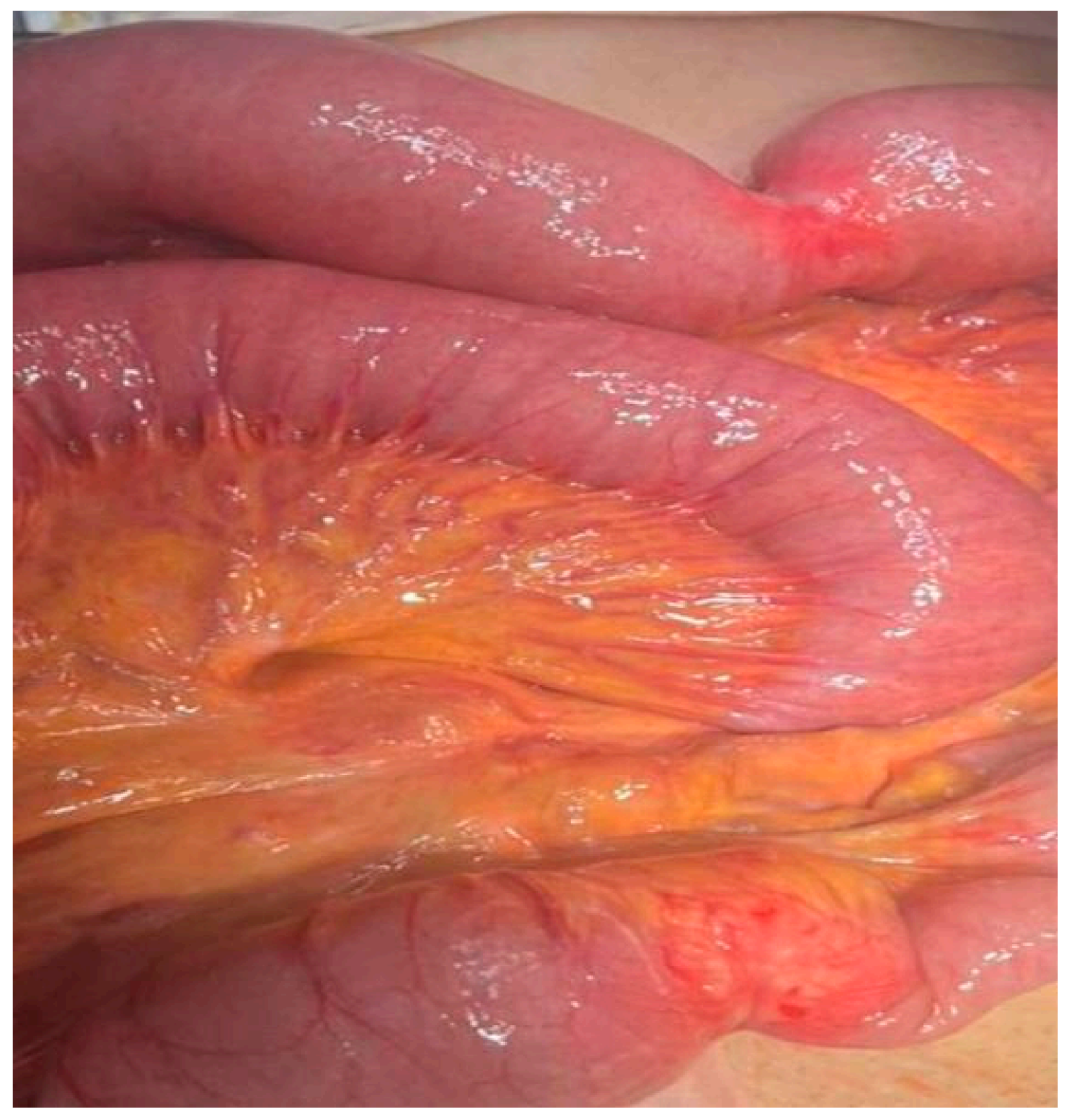
Intraoperative appearance of lymphoma (two tumors and multiple lymphadenopathies), suspected on CT and confirmed by histopathology and immunohistochemistry on the operative specimen (enterectomy with lymphadenectomy). Original intraoperative image from the authors’ own material, not previously published.

On CT, small-bowel lymphoma frequently presents as circumferential, relatively homogeneous wall thickening involving a longer intestinal segment, sometimes associated with aneurysmal dilatation of the lumen at the level of the tumor segment. Unlike adenocarcinoma, the transition between normal wall and the affected segment may be less abrupt, and severe stenosis with proximal loop dilatation is less characteristic. Mesenteric or retroperitoneal lymphadenopathy, sometimes multiple and bulky, is another important orienting element [22,29]. The radiological–histopathological correlation is supported by Bayoumi et al., who identified lymphadenopathy in all 13 analyzed lymphomas, whereas absence of proximal intestinal dilatation was present in 76.9% of cases [22]. More recently, Liu et al. showed that, compared with adenocarcinoma, lymphomas exhibited a larger tumor size and a higher degree of luminal preservation, as well as lower attenuation values in the arterial and venous phases [35]. Differentiating lymphoma from adenocarcinoma may be difficult, particularly when both present with circumferential wall thickening. Nevertheless, involvement of a longer intestinal segment, relative luminal preservation or aneurysmal dilatation of the tumor segment, and the presence of bulky mesenteric lymphadenopathy favor lymphoma (Figure 4), whereas a shorter, irregular, stenosing lesion associated with dilatation of the proximal loops is more suggestive of adenocarcinoma [22,29,35].

In complicated forms, this differentiation may become even more difficult. Tumor necrosis, perforation, peritumoral inflammation, fistulization, or obstruction may modify the usual morphology and reduce the discriminatory value of isolated CT signs. In this context, imaging interpretation must integrate several elements—the length of the affected segment, the character of the wall thickening, the degree of stenosis, the ratio between the lumen of the tumor segment and the proximal loops, and the distribution of lymphadenopathy—rather than rely on a single feature.

With respect to intraoperative assessment, lymphoma may present as circumferential or segmental thickening of the intestinal wall, sometimes involving a longer segment and without the pronounced retractile, stenosing character seen in adenocarcinoma. The lumen may remain relatively preserved, or the tumor segment may be dilated, and the presence of multiple, bulky mesenteric lymphadenopathies may constitute an additional element orienting toward a lymphoproliferative process. These findings are not, however, pathognomonic, and in perforated, necrotic, or intensely inflammatory forms the operative appearance may become difficult to differentiate both from other neoplasms and from inflammatory intestinal processes. Unlike CT features, for which radiological–histopathological studies demonstrate associations with a diagnosis of lymphoma [22,29,35], data quantifying the independent ability of the intraoperative macroscopic impression to predict the histological diagnosis are limited.

Consequently, the operative appearance must be integrated with imaging data and the clinical context and should not be considered the equivalent of the definitive histopathological diagnosis. On immediate macroscopic examination of the resection specimen, intestinal lymphoma may show an infiltrative, ulcerative, polypoid, or circumferential pattern, with wall thickening over a variable segment.

Unlike adenocarcinoma, circumferential involvement is not necessarily associated with severe stenosis, and the lumen may remain relatively wide or show aneurysmal dilatation. Thus, in a patient operated on without a preoperative histopathological diagnosis, the association of involvement of a relatively long intestinal segment, circumferential thickening with relative luminal preservation or aneurysmal dilatation of the tumor segment, and the presence of multiple mesenteric lymphadenopathies may support the suspicion of lymphoma [27].

### 4.4. Neuroendocrine Tumors (SBNET)

Small-bowel neuroendocrine tumors (SBNETs) exhibit a biological pattern distinct from that of adenocarcinoma, GIST, and lymphoma. The primary tumor is frequently small, submucosal, and predominantly located in the distal ileum, so that the size of the primary lesion may be disproportionately small compared with the extent of mesenteric disease. An important feature is multifocality, synchronous primary tumors being described in a substantial proportion of cases and potentially missed on preoperative imaging. The ENETS 2024 guideline reports multifocality in approximately 40–60% of patients and emphasizes the need for manual palpation of the entire small bowel during surgery [36]. Clinically, SBNETs may remain paucisymptomatic for a long time, and abdominal manifestations are often determined not only by the primary tumor but also by mesenteric lymphadenopathy and the associated desmoplastic reaction. Mesenteric fibrosis may cause retraction of the mesentery, angulation and fixation of the intestinal loops, impairment of the vascular supply and, in advanced forms, abdominal pain, subocclusive or occlusive phenomena, and intestinal ischemia. The desmoplastic reaction frequently develops around mesenteric nodal metastases and represents one of the important biological particularities of SBNETs [52].

On CT, the primary tumor may appear as a small hypervascular parietal or submucosal lesion, sometimes difficult to identify, particularly for subcentimeter tumors. In contrast, one of the most suggestive findings is a mesenteric nodal mass associated with a desmoplastic reaction, with fibrosis and retraction of the mesentery and adjacent intestinal loops. Mesenteric vascular involvement may become important both for symptomatology and for assessing resectability. Recent surgical literature emphasizes precisely the importance of preoperative assessment of nodal extension, mesenteric fibrosis, and the relationships with the mesenteric vessels [43]. These features are also valuable in orienting the histological diagnosis. In the radiological–pathological study by Bayoumi et al., mesenteric changes were identified in all 14 analyzed carcinoid/NET cases (100%; *p* = 0.001), representing the imaging feature significantly associated with this histology [22]. However, this association should not be interpreted as a high sensitivity of CT for identifying the primary tumor. In SBNETs, mesenteric signs may be evident even when the primary intestinal lesion is small or remains occult on imaging [16,20]. Deguelte et al. explicitly describe small primary tumors, sometimes undetectable, associated with a mesenteric nodal mass and a desmoplastic reaction, and the ESES consensus indicates that occult primaries are frequently small and multifocal [20].

**Figure 5:**
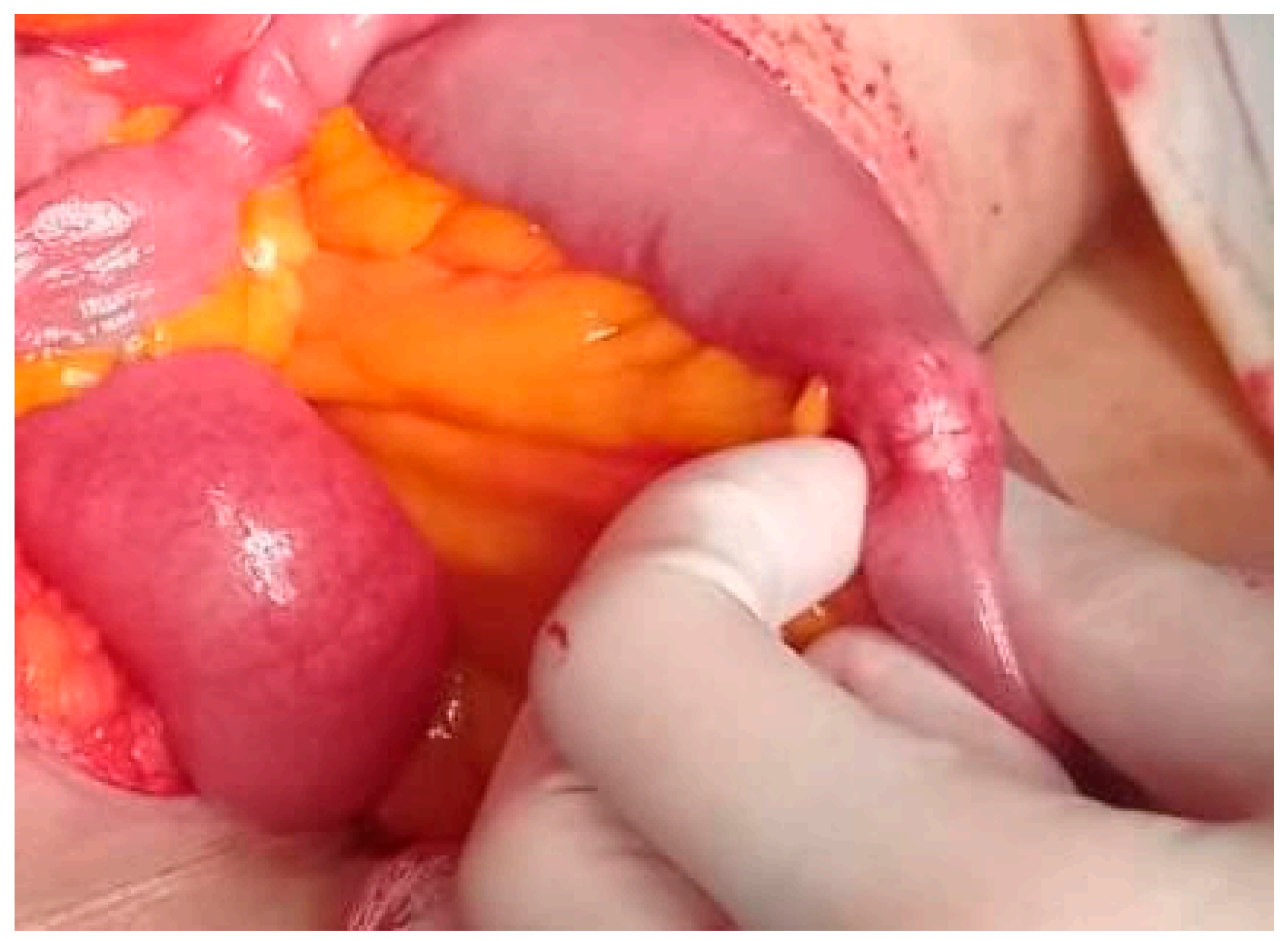
Intraoperative appearance of a SBNET (not suspected on CT; confirmed by histopathology and immunohistochemistry). Original intraoperative image from the authors’ own material, not previously published.

Intraoperative assessment is of particular importance in SBNETs, because the primary tumors are frequently small, submucosal, and multifocal and may have a discreet or even apparently normal serosal appearance. Consequently, simple inspection of the bowel may be insufficient, and systematic manual palpation of the entire small bowel is an essential element for identifying primary lesions and multifocality. Recent data confirm this limitation of preoperative imaging: in a study of 104 patients, histopathological examination demonstrated multifocality in 51% of cases, whereas ^68^Ga-DOTATATE PET identified it preoperatively in only 27%, with a sensitivity of 45% [33]. This limitation is particularly relevant for subcentimeter and multifocal lesions, in which the serosal surface may be discreetly altered or apparently normal, so that palpation may provide additional information beyond simple intraoperative inspection [16,20]. The relationship between preoperative imaging and surgical exploration is therefore particular in SBNETs. CT can characterize the mesenteric nodal mass, the desmoplastic reaction, and its relationships with the mesenteric vessels, whereas intraoperative exploration—through inspection and, above all, manual palpation—can identify small or multifocal primary tumors not detected preoperatively (Figure 5). The two modalities should be regarded as complementary, each providing different information relevant to planning and to the extent of resection [43,44].

On macroscopic examination of the resection specimen, SBNETs may present as small intramural or submucosal nodules, sometimes multiple, distributed along the resected intestinal segment. Careful examination and palpation of the specimen may provide additional information, particularly in multifocal forms, by identifying and characterizing lesions that may be difficult to detect by simple inspection of the serosal surface. Studies performed on resection specimens have demonstrated a variable number of synchronous primary tumors, sometimes numerous, supporting the importance of evaluating the entire intestinal segment [53,54]. Nevertheless, macroscopic examination has important limitations: microscopic, nonpalpable neuroendocrine foci may exist even in apparently uninvolved segments, so that the macroscopic appearance of the specimen allows neither complete identification of multifocality nor definitive determination of the histological nature of the lesions, the definitive diagnosis remaining dependent on histopathological and immunohistochemical examination [55].

### 4.5. Desmoid-Type Fibromatosis

Desmoid-type fibromatosis is a rare fibroblastic proliferation without metastatic potential but with a capacity for local infiltration and recurrence, which intra-abdominally involves predominantly the mesentery and may secondarily involve the intestinal loops and adjacent vascular structures. Unlike adenocarcinoma, GIST, lymphoma, and SBNET, the biological problem is not metastatic dissemination but locally infiltrative behavior, sometimes disproportionate to the benign/intermediate histological character of the lesion. The contemporary management of desmoid tumors has changed significantly, with active surveillance being preferred in many asymptomatic or stable cases, and intervention being individualized according to symptoms, progression, location, and the anticipated morbidity of treatment [18].

Clinically, intra-abdominal desmoid-type fibromatosis may remain asymptomatic until its size or local extension causes abdominal pain, compression, transit disturbances, or intestinal obstruction. In bulky mesenteric forms, the tumor may encase or tether the intestinal loops and may have close relationships with the mesenteric vessels, so that symptomatology and surgical difficulty are determined more by local extension than by the size of any intestinal parietal component [56,57,58].

On CT, mesenteric desmoid-type fibromatosis usually presents as a solid mesenteric mass, well or poorly delineated, with density and degree of contrast enhancement varying according to the proportion of cellular, myxoid, and collagenous components. A suggestive feature is radiating or spiculated extensions into the adjacent mesenteric fat, which may confer a characteristic “whorled” appearance [21]. Necrosis is generally absent, and contrast enhancement may be homogeneous or heterogeneous and of variable intensity. Local extension may cause traction, displacement, or encasement of the intestinal loops and close relationships with the mesenteric vessels [21,59]. Nevertheless, the CT appearance of desmoid-type fibromatosis is not specific. Well-delineated lesions may mimic GIST or other mesenteric tumors, whereas infiltrative forms may suggest a malignant neoplasm. In this context, the association of a mesenteric origin, radiating extensions into the adjacent fat, absence of necrosis, and encasement or traction of the intestinal loops may increase the imaging suspicion of desmoid-type fibromatosis, without these features being pathognomonic [21,56,59].

**Figure 6:**
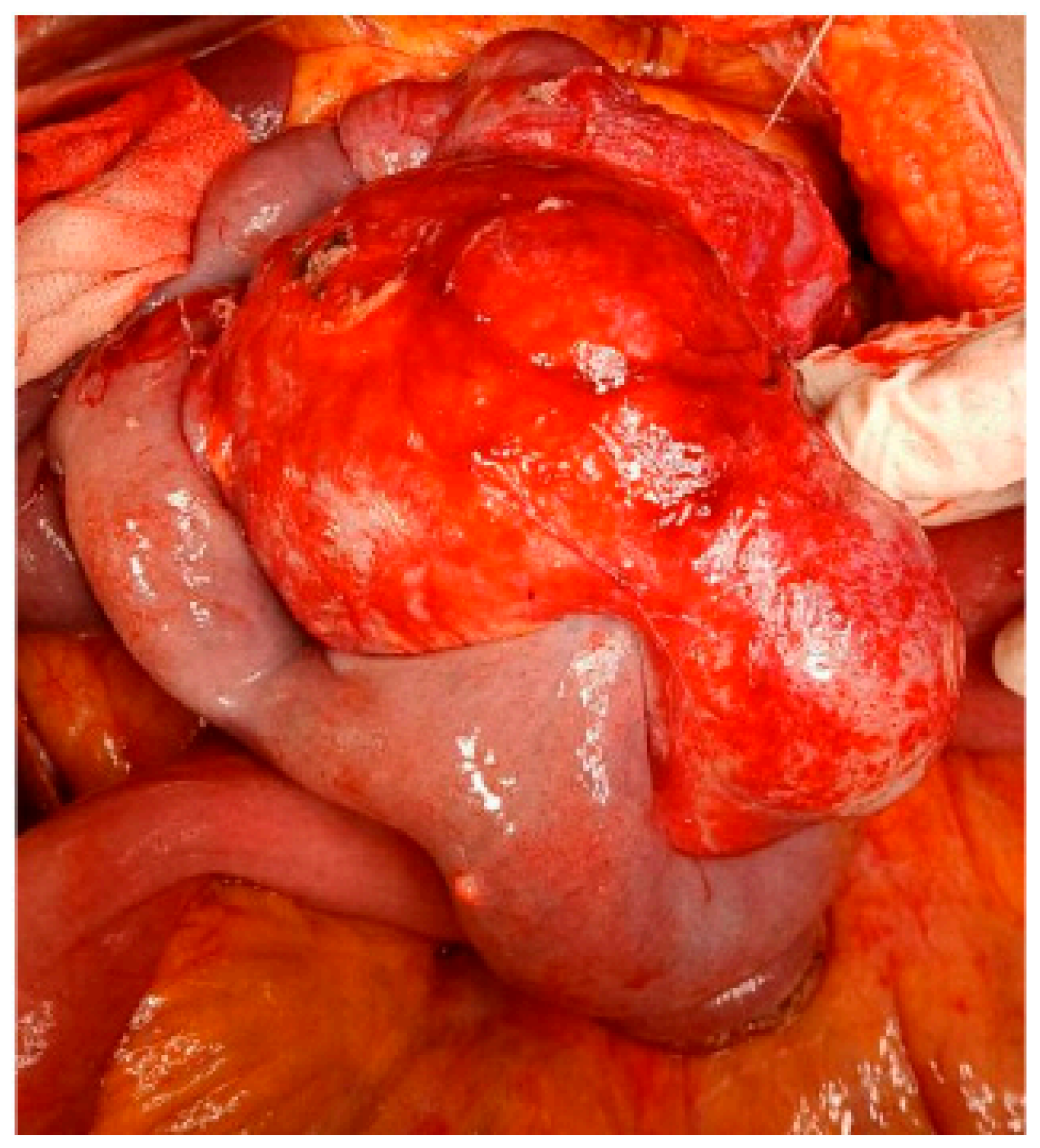
Intraoperative appearance of desmoid-type fibromatosis (inconclusive preoperative biopsy; confirmed postoperatively by histopathology and immunohistochemistry). Original intraoperative image from the authors’ own material, not previously published.

Intraoperatively, mesenteric desmoid-type fibromatosis may have a variable appearance, ranging from a relatively well-delineated formation to a firm, poorly delineated, infiltrative mass arising in the mesentery. The lesion may adhere to or infiltrate the intestinal loops and may cause mesenteric retraction, encasement of intestinal segments, and close relationships with the vascular pedicles (Figure 6). These macroscopic features are not specific and may erroneously suggest a malignant neoplasm. Cases have been described in which the intraoperative appearance of a mesenteric mass infiltrating the intestinal wall was interpreted as suggestive of GIST, the diagnosis of desmoid-type fibromatosis being established only on histopathological examination [26,45,60]. In this context, when the CT and/or intraoperative appearance raises the suspicion of desmoid-type fibromatosis and the clinical situation allows the definitive intervention to be deferred, obtaining a histopathological diagnosis by image-guided core biopsy should be considered before extensive resection. This strategy is applicable particularly to stable patients or to cases in which a possible complication can be temporarily controlled, whereas complete obstruction, perforation, intestinal ischemia, or uncontrolled hemorrhage generally mandate surgical treatment without the possibility of deferral for histological confirmation [61,62,63,64]. Frozen-section examination may be considered when the diagnosis remains uncertain intraoperatively, but its value for differentiating desmoid-type fibromatosis from other spindle-cell tumors is insufficiently validated, the definitive diagnosis frequently requiring immunohistochemistry and, in selected cases, molecular analysis [64].

Thus, in a patient without a preoperative histopathological diagnosis, identification of an infiltrative mesenteric mass with imaging features suggestive of desmoid-type fibromatosis should prompt caution before an extensive intestinal or mesenteric resection. When the clinical situation allows treatment to be deferred, histopathological confirmation by image-guided core biopsy may avoid a procedure with important morbidity for a lesion whose contemporary management is not necessarily surgical. Conversely, in the presence of a complication requiring immediate treatment—such as perforation, ischemia, complete obstruction, or uncontrolled hemorrhage—the priority of the surgical procedure remains control of the complication, and the extent of resection should be adapted to the intraoperative context and to the need to preserve bowel [28,65].

## 5. Surgical Decision-Making When Histology Is Unknown

When preoperative histological confirmation is unavailable, surgical decision-making should not rely on a single radiological or intraoperative feature. Rather, the clinical context, imaging characteristics, intraoperative findings, disease extent, and resectability should be interpreted together. CT features may orient the differential diagnosis [22,29,35,38], while intraoperative exploration can reveal multifocal disease, mesenteric fibrosis, occult lesions, or the true relationship of the mass to adjacent structures [16,20,33,43]. None of these features is sufficiently specific to replace definitive histopathological examination.

The implications are histology-dependent. A short, irregular, stenosing lesion with proximal dilatation may support a working diagnosis of adenocarcinoma and favor oncological segmental resection with mesentery and regional lymph nodes [22,23,25,29]. A well-delineated exophytic mass without significant regional lymphadenopathy may favor GIST, for which complete resection with preservation of tumor integrity and avoidance of rupture are central and routine lymphadenectomy is generally unnecessary [14,22,32]. SBNET should be suspected when a small or occult ileal primary is associated with a mesenteric nodal mass, desmoplastic reaction, fibrosis, and/or vascular involvement; systematic exploration and palpation of the entire small bowel are important because preoperative imaging may underestimate multifocal disease [16,20,33,36,43].

Lymphoma requires particular caution. In the absence of obstruction, perforation, hemorrhage, ischemia, or another complication that independently mandates surgery, obtaining tissue before resection is preferred because a systemic diagnosis may permit non-surgical treatment and may avoid an unnecessary oncological intestinal resection [17,27,31]. When an acute complication is present, the operative objective is instead dictated by control of the complication, with resection tailored to restore continuity or control contamination/bleeding while preserving bowel when feasible [17,41].

Desmoid-type fibromatosis represents another situation in which diagnostic uncertainty can alter management substantially. A stable patient with an infiltrative mesenteric mass and imaging features compatible with desmoid tumor should generally undergo multidisciplinary assessment and, when treatment can safely be deferred, image-guided core biopsy before extensive resection [18,21,28,45]. Conversely, complete obstruction, perforation, ischemia, or uncontrolled hemorrhage may require immediate surgery, with the extent of resection adapted to the complication and the need to preserve bowel [28,61,65].

**Figure 7:**
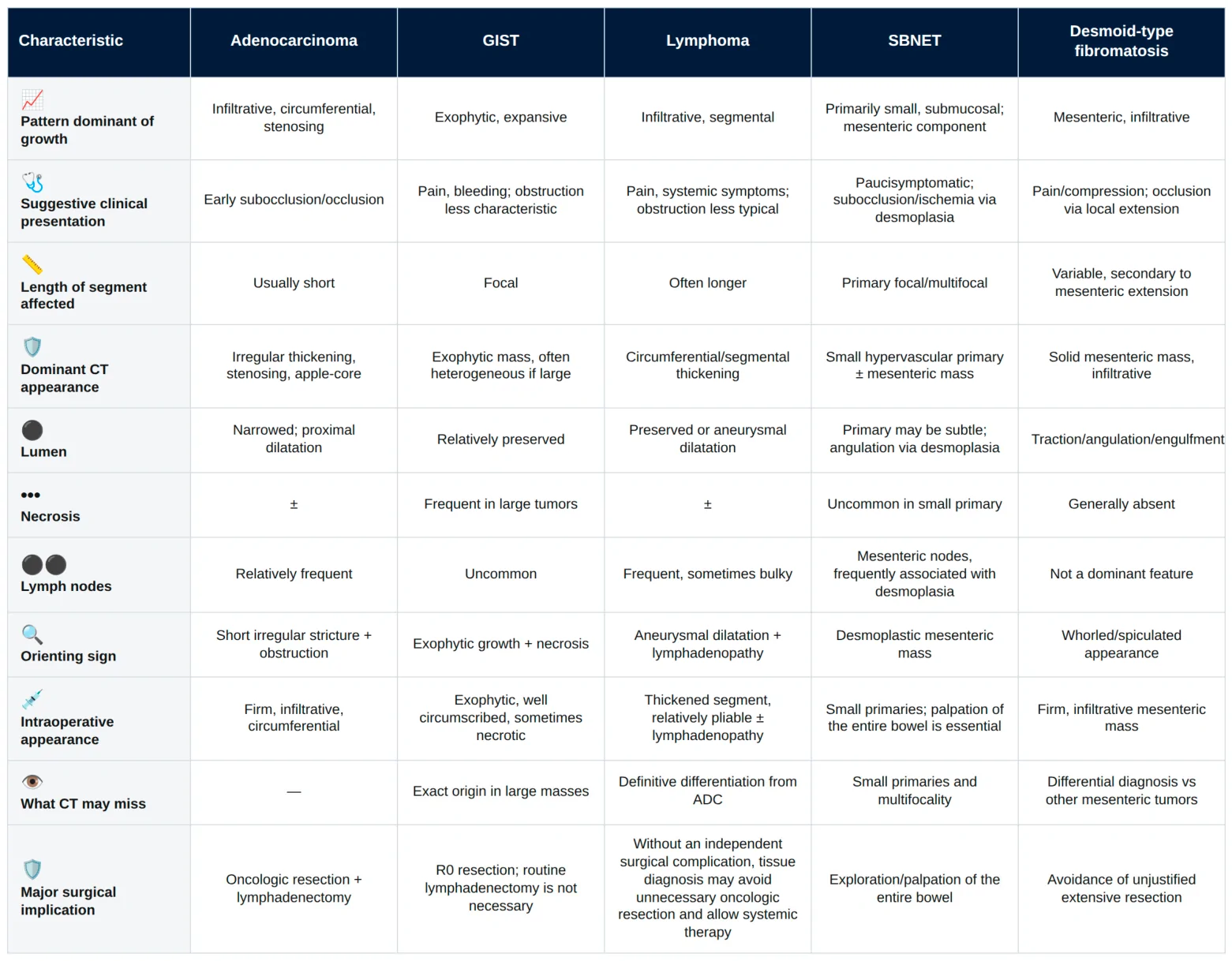
Comparative features of the main small-bowel tumor types relevant to presumptive diagnosis and surgical decision-making. No imaging or intraoperative characteristic should be interpreted as diagnostic in isolation. Source: authors’ synthesis based on the studies cited in the manuscript.

Based on these differential diagnostic features, we propose a pragmatic framework that prioritizes, in sequence: (1) clinical urgency; (2) disease extent and resectability; (3) whether preoperative tissue can be obtained safely and whether the result would change management; and (4) the likely consequences of proceeding without histology. The framework is a synthesis derived from the evidence analyzed in this review and is conceptual rather than a prospectively validated clinical prediction model [14,16,18,20,22,28,29,32,33,36,43]. It is intended to support structured reasoning under diagnostic uncertainty rather than to establish histology intraoperatively. A comparative summary of the main distinguishing clinical, imaging, and intraoperative features across tumor types is presented in Figure 7.

Importantly, the proposed histology-oriented surgical reasoning is primarily applicable to patients with potentially resectable disease, in whom the presumed tumor type may directly influence the extent and oncological principles of resection. In patients with extensive metastatic disease or peritoneal carcinomatosis presenting with acute complications such as obstruction, hemorrhage, or perforation, the operative objective may instead be dictated by control of the life-threatening complication, and a formal oncological resection may not always be appropriate.

**Figure 8:**
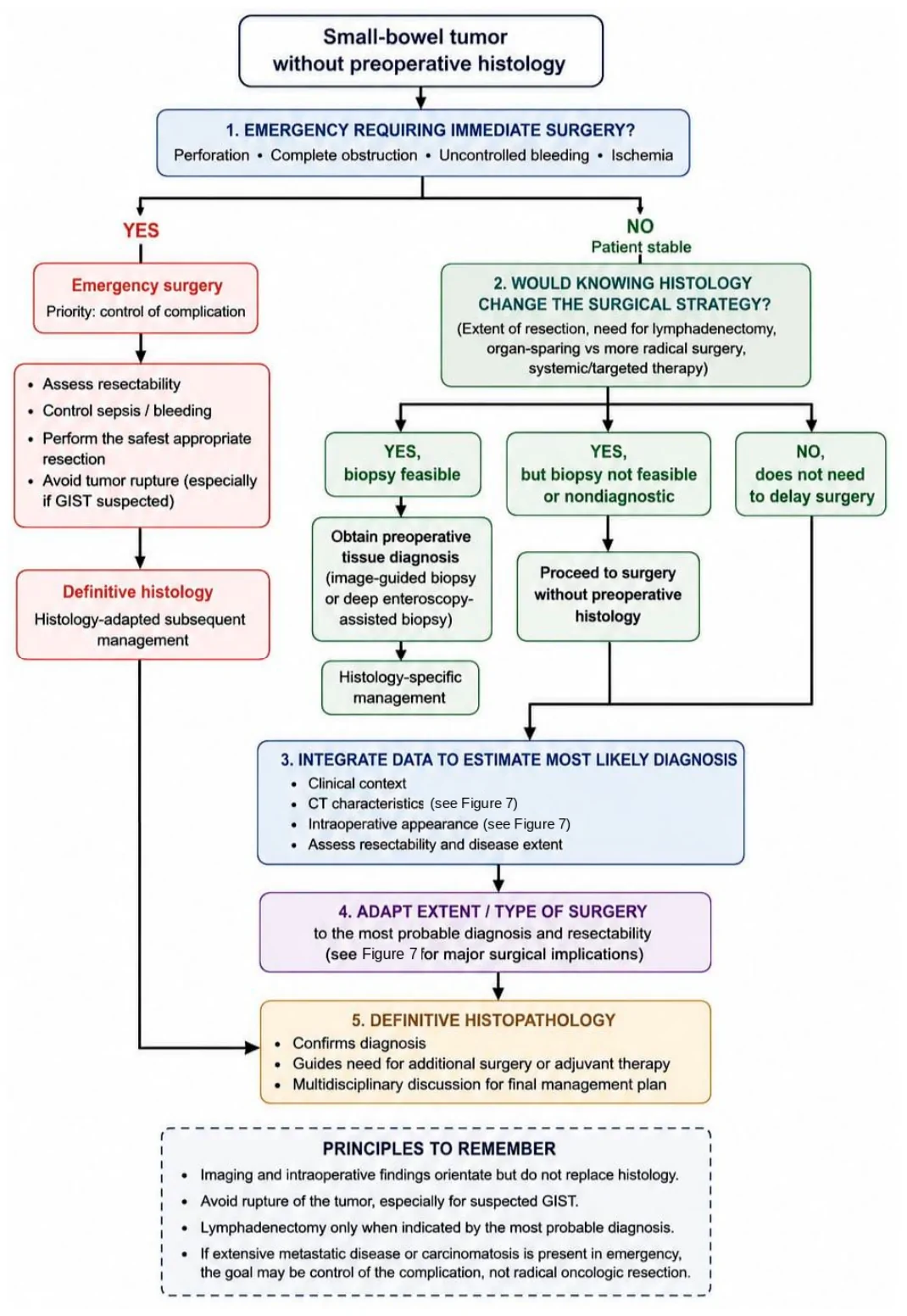
Proposed decision-making framework for small-bowel tumors without preoperative histological confirmation. The algorithm is intended for patients in whom there is already an operative indication or surgery is actively being considered; for suspected lymphoma or desmoid-type fibromatosis, preoperative tissue acquisition remains preferred whenever the clinical situation permits. Source: authors’ synthesis based on the studies cited in the manuscript.

Based on these differential diagnostic features, the next step is to translate the presumptive diagnosis into a practical surgical decision. Rather than attempting to predict histology with certainty, the proposed decision-making framework for patients with small-bowel tumors in whom preoperative histology is unavailable prioritizes clinical urgency, disease extent and resectability, the feasibility of obtaining tissue before surgery, and the potential impact of histology on the extent of the surgical procedure (Figure 8).

## 6. Limitations

The main limitation of the available evidence is its heterogeneity and uneven distribution across histological subtypes. While correlations between CT features and final histopathology are relatively well documented, evidence regarding the independent ability of intraoperative macroscopic findings to predict histology remains considerably more limited. Histology-specific imaging associations derive in part from relatively small retrospective cohorts and should therefore not be interpreted as universally applicable estimates of diagnostic sensitivity or specificity.

Imaging performance may be influenced by tumor size and location, CT protocol, bowel distension, and acute complications. Obstruction, perforation, ischemia, hemorrhage, or associated inflammatory changes may modify otherwise characteristic radiological and macroscopic patterns, further limiting reliable histological prediction in precisely those patients in whom urgent surgical decisions may be required. Moreover, the molecular heterogeneity reported in gastrointestinal adenocarcinomas further illustrates the biological complexity that may exist even among tumors with similar histopathological classifications [12,13].

The role of intraoperative biopsy and frozen-section examination is also insufficiently standardized. In particular, differentiation between desmoid-type fibromatosis and other spindle-cell tumors may require immunohistochemistry and, in selected cases, molecular analysis; consequently, intraoperative morphology or frozen-section examination cannot always provide a definitive diagnosis. Finally, the surgical decision-making framework proposed in this review is conceptual and has not been prospectively validated. It should therefore be regarded as a structured aid for surgical reasoning under diagnostic uncertainty rather than as a substitute for histopathological diagnosis. Future prospective and preferably multicenter studies should systematically correlate preoperative imaging, intraoperative findings, final histology, and the resulting surgical strategy to determine whether this integrated approach can reduce both oncologically inadequate and unnecessarily extensive resections.

## 7. Future Directions

The present review highlights an important gap that extends beyond emergency surgery and is also relevant to elective cases in which preoperative histology cannot be obtained. Traditionally, when a tissue diagnosis was unavailable, the surgeon often relied predominantly on intraoperative macroscopic findings to determine the extent and oncological character of resection. However, current evidence suggests that histology-specific correlations are better documented for preoperative imaging than for the surgeon’s macroscopic impression alone.

Future decision-making should therefore move from an essentially intraoperative approach toward an integrated radiological–surgical assessment, in which the CT phenotype and intraoperative findings are interpreted together rather than sequentially or independently. Tissue confirmation remains desirable whenever it can be obtained safely, but its feasibility and diagnostic yield vary according to tumor location and histology. Percutaneous core biopsy is more applicable to accessible solid or extraluminal masses, whereas sampling of intramural lesions of a hollow viscus may be technically more difficult and may carry procedural risks. Similarly, frozen-section examination may provide useful information in selected cases but cannot resolve all diagnostic uncertainties, particularly in the differential diagnosis of mesenchymal and spindle-cell lesions, for which definitive classification may require immunohistochemistry and, in selected cases, molecular characterization [32].

These limitations support a future research direction centered on the prospective validation of integrated diagnostic algorithms combining clinical presentation, CT phenotype, and intraoperative findings. Such studies should determine not only diagnostic accuracy for final histology but, more importantly, whether integration of radiological and surgical information can appropriately modify the extent of resection and reduce both oncologically inadequate and unnecessarily extensive procedures. This approach may be particularly valuable in emergency surgery, where a tissue diagnosis is frequently unavailable, but also in elective patients in whom preoperative biopsy is technically unfeasible or potentially unsafe.

## 8. Conclusions

Small-bowel tumors represent a surgical challenge when a preoperative histopathological diagnosis is unavailable. Integrating the clinical context, CT features, and intraoperative findings may support a presumptive diagnosis and help the surgeon determine the appropriate extent and oncological approach of resection. Preoperative imaging and intraoperative exploration should be regarded as complementary rather than competing approaches. Histopathological examination remains the definitive diagnostic standard; the purpose of this integrated approach is not to replace it, but to support safer surgical decision-making under diagnostic uncertainty and to reduce the risk of both oncologically inadequate and unnecessarily extensive resections.

## Figures and Tables

**Table 1:** (**a**) Included reviews and evidence summaries (2020–2026). (**b**) Included guidelines and consensus documents (2020–2026). (**c**) Included studies with original clinical data or case-based evidence (2020–2026).

**(a)**			
**Study**	**Design**	**Tumor/Focus**	**Key Contribution Relevant to Decision-Making**
Deguelte et al. (2020) [20]	Review	SBNET	Small/multifocal ileal primaries; mesenteric nodal disease and desmoplastic reaction; systematic palpation emphasized.
Kim (2020) [6]	Review	Small-bowel tumors	Capsule endoscopy and DAE are complementary; DAE is important when tissue confirmation is required.
Rosa et al. (2020) [21]	Pictorial review	Desmoid-type fibromatosis	CT/MRI may show infiltrative margins and radiating/whorled extensions; imaging supports differential diagnosis and extent assessment.
Shinya (2022) [9]	Review	Malignant small-bowel neoplasms	Tumor-specific CT enhancement and morphology can narrow the differential, but histological classification remains difficult.
Khosla et al. (2022) [23]	Overview	Small-bowel adenocarcinoma	Multimodality diagnosis; complete resection with negative margins and lymph-node dissection remains central.
Inoue et al. (2022) [24]	Radiological review	GIST	Exophytic growth, heterogeneous enhancement, necrosis, hemorrhage, and cystic degeneration are typical imaging clues.
Turpin et al. (2022) [25]	Review/evidence summary	Localized SBA	Oncologic resection with adequate margins, mesentery, and regional lymph nodes; evidence for perioperative therapy remains limited.
Khdhir et al. (2022) [27]	Clinical/imaging review	Abdominal lymphoma	Long-segment wall involvement, relative luminal preservation/aneurysmal dilatation, and lymphadenopathy; complications can dominate presentation.
Vlachou et al. (2023) [30]	Narrative review	Small-bowel tumors	Cross-sectional imaging and advanced enteroscopy have complementary roles in detection, characterization, and tissue acquisition.
Lee et al. (2023) [29]	Radiologic review	SBA, GIST, lymphoma, SBNET	Characteristic CT growth patterns may guide presumptive diagnosis when preoperative histology is unavailable.
Yano and Yamamoto (2024) [7]	Review	Small-bowel tumors	Three-phase enhanced CT is emphasized as first-line; DAE/BAE can provide biopsy and localization when feasible.
Brogna et al. (2025) [38]	Pictorial review	Benign/malignant intestinal tumors	CT is central, particularly in emergencies, for characterization, staging, and treatment planning.
Hallet et al. (2026) [43]	Expert surgical review	SBNET	Surgical planning integrates imaging with mesenteric nodal disease, fibrosis, vascular involvement, and systematic bowel exploration.
Xia et al. (2026) [44]	Systematic review/meta-analysis	Primary small-bowel tumors	Diagnostic performance varies by modality and tumor characteristics; imaging should be integrated with clinical/intraoperative information.
**(b)**			
**Scheme**	**Design**	**Tumor/Focus**	**Key Contribution Relevant to Decision-Making**
Casali et al. (2022) [14]	ESMO/EURACAN/GENTURIS guideline	GIST	R0 resection while avoiding rupture; routine lymphadenectomy not indicated for clinically node-negative disease; biopsy when diagnosis would alter approach or systemic therapy is contemplated.
Benech et al. (2022) [28]	Clinical practice guideline	Desmoid-type fibromatosis	Individualized multidisciplinary management; active surveillance appropriate in selected patients; upfront surgery is not systematic.
Pennazio et al. (2023) [31]	ESGE guideline	Small-bowel disorders/tumors	Capsule endoscopy and DAE are complementary; DAE permits targeted biopsy when accessible and technically feasible.
Serrano et al. (2023) [32]	GEIS guideline	GIST	Complete resection with preserved tumor integrity; systematic lymphadenectomy generally unnecessary.
Van Den Heede et al. (2024) [16]	ESES consensus	Advanced/metastatic SBNET	Occult primaries may be small and multifocal; exploration should not depend on successful preoperative localization.
Lamarca et al. (2024) [36]	ENETS guidance	Well-differentiated SBNET	Systematic exploration/palpation and assessment of mesenteric nodal disease are emphasized.
Kasper et al. (2024) [18]	International consensus review/update	Desmoid tumor	Management increasingly favors less invasive strategies based on symptoms, location, progression, morbidity, and patient preference.
**(c)**			
**Study**	**Design**	**Tumor/Focus**	**Key Contribution Relevant to Decision-Making**
Bayoumi et al. (2021) [22]	Retrospective radiological-pathological	Adenocarcinoma/GIST/lymphoma/SBNET	Irregular/asymmetric wall thickening associated with adenocarcinoma; exophytic growth with GIST; lymphadenopathy/luminal preservation with lymphoma; mesenteric changes with SBNET.
Vashistha et al. (2022) [10]	Retrospective single-center	Small-bowel tumors	Segmental resection for most sites; pancreatoduodenectomy for selected second-part duodenal tumors; adequate lymph-node harvest.
Hajri et al. (2022) [26]	Case report	Mesenteric desmoid-type fibromatosis	Emergency en bloc resection in a perforated/infiltrative presentation; final diagnosis required histopathology/IHC.
Dolu et al. (2023) [4]	Retrospective	Small-bowel tumors	DBE diagnosed 59.5% of mass-like lesions histologically; 40.5% required surgical diagnosis.
Zhang et al. (2023) [33]	Retrospective, *n* = 104	SBNET	Histopathology found multifocality in 51%, versus 27% detected by 68Ga-DOTATATE PET; supports systematic intraoperative exploration.
Obleagă et al. (2024) [2]	10-year retrospective observational	Primary resectable small-bowel cancers	CT, local invasion, nodes, and multiplicity influenced surgery; definitive diagnosis by histopathology/IHC; no specific histology-linked macroscopic pattern established.
Hasnaoui et al. (2024) [34]	Case report	Locally advanced jejunal GIST	Large locally advanced tumor treated by en bloc multivisceral resection; final GIST confirmed by histopathology/IHC.
Liu et al. (2024) [35]	Retrospective diagnostic study	Small-intestinal tumors/tumor-like lesions	CT enterography differentiated malignant from nonmalignant lesions; lymphomas showed larger size and greater luminal preservation than adenocarcinoma.
Cazacu et al. (2025) [1]	Retrospective cohort, *n* = 80	Benign/malignant small-bowel tumors	72.5% malignant; CT detected tumors in 81.3% of imaged patients; surgical approach varied from radical resection to biopsy/endoscopic treatment.
Torres-Jurado et al. (2025) [17]	Retrospective prospective-database study, *n* = 17	Complicated primary intestinal lymphoma	Emergency surgery was driven by obstruction, perforation, or hemorrhage; no validated macroscopic pattern distinguished lymphoma.
Li et al. (2025) [37]	Retrospective diagnostic, *n* = 129	SIST/GIST vs. primary intestinal lymphoma	CECT morphologic and texture features improved differentiation; combined model AUC 0.927 vs. 0.847 for CECT features alone.
Fujimura et al. (2025) [39]	Case report	Ileal DLBCL	Wall thickening, obstruction, and mesenteric nodes on CT preceded segmental resection; final diagnosis by pathology/IHC.
Bist and Ghimire (2026) [40]	Retrospective case series, *n* = 6	Small-intestinal GIST	Varied exophytic/intraluminal and perforated presentations; segmental or wedge resection according to site.
Gao et al. (2026) [41]	Case report	Jejunal T-cell lymphoma/EATL	Perforation required emergency resection; definitive diagnosis depended on postoperative histopathology/IHC.
Sbitan et al. (2026) [42]	Case report/focused review	Ileal adenocarcinoma	Pelvic mass mimicking gynecologic malignancy; segmental ileal resection with regional lymphadenectomy and gynecologic surgery.
Tokumaru et al. (2026) [45]	Case report	Mesenteric desmoid	Desmoid mimicked malignant small-bowel tumor on CT; no preoperative biopsy; diagnostic uncertainty influenced extent/strategy of resection.

Abbreviations: DAE, device-assisted enteroscopy; BAE, balloon-assisted enteroscopy; DBE, double-balloon enteroscopy; CTE, CT enterography; CECT, contrast-enhanced CT; MDCT, multidetector CT; IHC, immunohistochemistry; SBNET, small-bowel neuroendocrine tumor; SBA, small-bowel adenocarcinoma.

## Data Availability

No new data were created or analyzed in this study. Data sharing is not applicable to this article.
